# Testosterone Therapy as an Isolated Risk Factor for Venous Thrombosis: A Case Report

**DOI:** 10.7759/cureus.63611

**Published:** 2024-07-01

**Authors:** Kara Bragg, Hailey Miller, Ricky Buckshaw, Leslie V Simon, Courtney King

**Affiliations:** 1 Emergency Medicine, Mayo Clinic, Jacksonville, USA; 2 Internal Medicine, Mayo Clinic, Jacksonville, USA; 3 Emergency Medicine/Medical Simulation, Mayo Clinic, Jacksonville, USA

**Keywords:** wells' criteria, pulmonary embolism rule-out criteria (perc), case report, testosterone, venous thrombus embolism

## Abstract

Healthcare providers in the emergency department (ED) diagnose and treat venous thrombus embolism (VTE). Current VTE clinical decision tools specifically mention estrogen but do not include the use of testosterone replacement therapy (TRT). A male in the early third decade of life presented to the ED with complaints of left calf pain and subjective swelling for one week. The patient was low risk using VTE scoring tools. The patient had multiple occlusive VTE on lower-extremity ultrasound and extensive central, bilateral segmental, and subsegmental pulmonary emboli. He was admitted to the hospital and placed on a heparin drip. TRT may be seen in clinical practice in both male and female patients. This bodes the question of whether TRT should be added to the risk analysis tools used to evaluate and predict the risk for VTE. There have been conflicting reports in research, with more recent studies indicating the risk associated with TRT. This case study demonstrates the risk of VTE with TRT. Further investigation is needed to determine if there is a correlation between VTE and TRT. Clinical decision tools should be updated to include TRT accordingly.

## Introduction

Testosterone therapy has been used to treat diminished libido, erectile dysfunction, and testosterone deficiency and to improve physical strength and endurance [1]. Testosterone prescriptions have increased over the past decades, up to 300% from 2001 to 2013 [2]. Despite warnings from the Federal Drug Administration (FDA) in 2015 on the association of testosterone with venous thrombus embolism (VTE), prescriptions continued to rise again with an average of 8% increase in prescribers from 2016 to 2019. There have been studies over the last few decades with inconsistent conclusions regarding risks associated with testosterone use in men with and without hypogonadism. This bodes the question of how patients should be evaluated for VTE risk in the emergency department (ED). The Wells' criteria for deep venous thrombosis (DVT) and pulmonary embolism (PE) do not mention hormone therapy [3]. The Four-Level Pulmonary Embolism Clinical Probability Score (4PEPS) mentions estrogen use [4], and the PERC score broadens the scope to hormone replacement or use of estrogen [5]. The YEARS criteria evaluate the probability of PE and include pregnancy as a hormone component [4]. There is currently no risk analysis score that considers testosterone replacement therapy. The following case report shows the need for additional research into the incorporation of testosterone, including that prescribed for replacement or hypogonadism, into our risk analysis for patients in the ED.

## Case presentation

A male in his early third decade of life presented to the ED for evaluation of pain and swelling in the left lower extremity. The pain had been present the last two weeks with significant exacerbation over the last week. He denied injury, overuse, redness, fever, or chills. He denied any recent travel, prior injury, surgery, prolonged periods of immobility, cancer, estrogen therapy, or coagulation disorders. He reported subjective swelling in his left calf, although no significant increase in size. The patient was tachycardic at 120 on arrival to triage, but the sinus rhythm was in the 90s at rest. The respiratory rate was 18 even and unlabored, and oxygen saturation was 98%. On further review of systems, the patient did state that he was feeling mild shortness of breath and noted that his heart rate was high at times when he was exerting himself, but attributed this to the pain he experienced in his calf while going up the stairs. On exam, he was found to be tender in the medial aspect of his left calf without appreciable swelling greater than 3 cm. His sister who was a registered nurse prompted him to come in to be evaluated secondary to family history. He reported that his father had a blood clot in his leg at age 70. He reported no other medical history, and he denied taking any medications when he presented to the ED.

**Figure 1:**
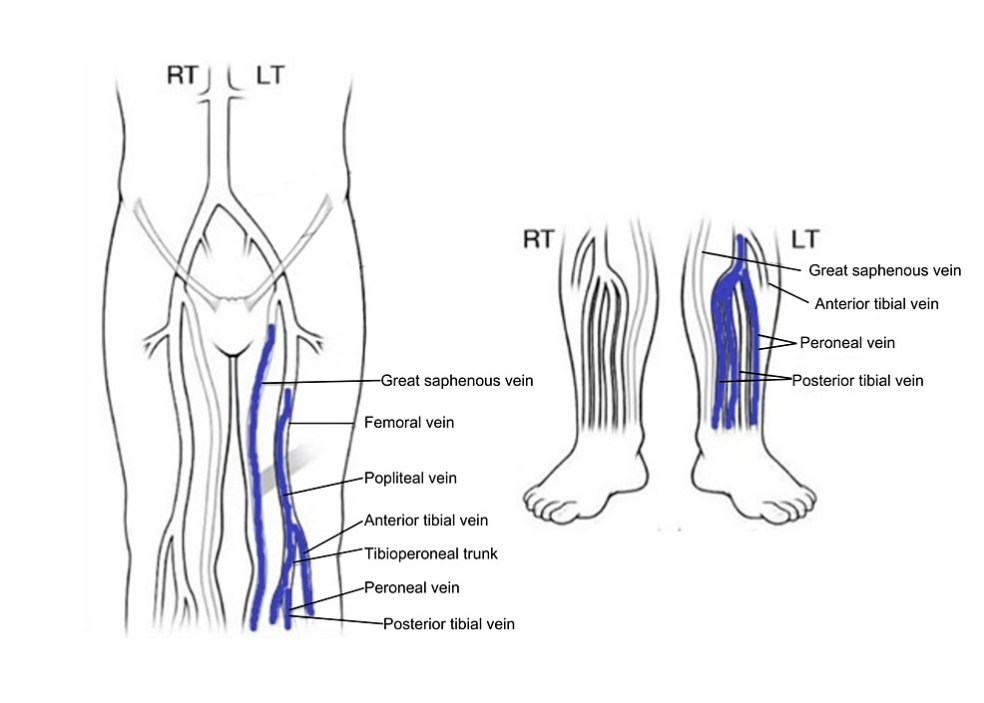
Ultrasound Doppler study showing multiple deep vein thrombosis. There was a partially occlusive thrombus in the femoral vein mid thigh and fully occlusive thrombus in the distal femoral vein to the popliteal vein. There was an occlusive thrombus in the calf vein. Image reproduced from radiology images obtained on a deep vein thrombus (DVT) study. Mayo Clinic is the copyright holder of the DVT mapping image.

An ultrasound was ordered after the initial assessment in the triage area while the patient was waiting to be roomed. The ultrasound was returned showing acute deep vein thrombosis (DVT) involving left mid to lower femoral, popliteal, posterior tibial, and peroneal veins with superficial venous thrombus (SVT) of the greater saphenous vein from the upper thigh to the calf (Figure 1).

**Figure 2:**
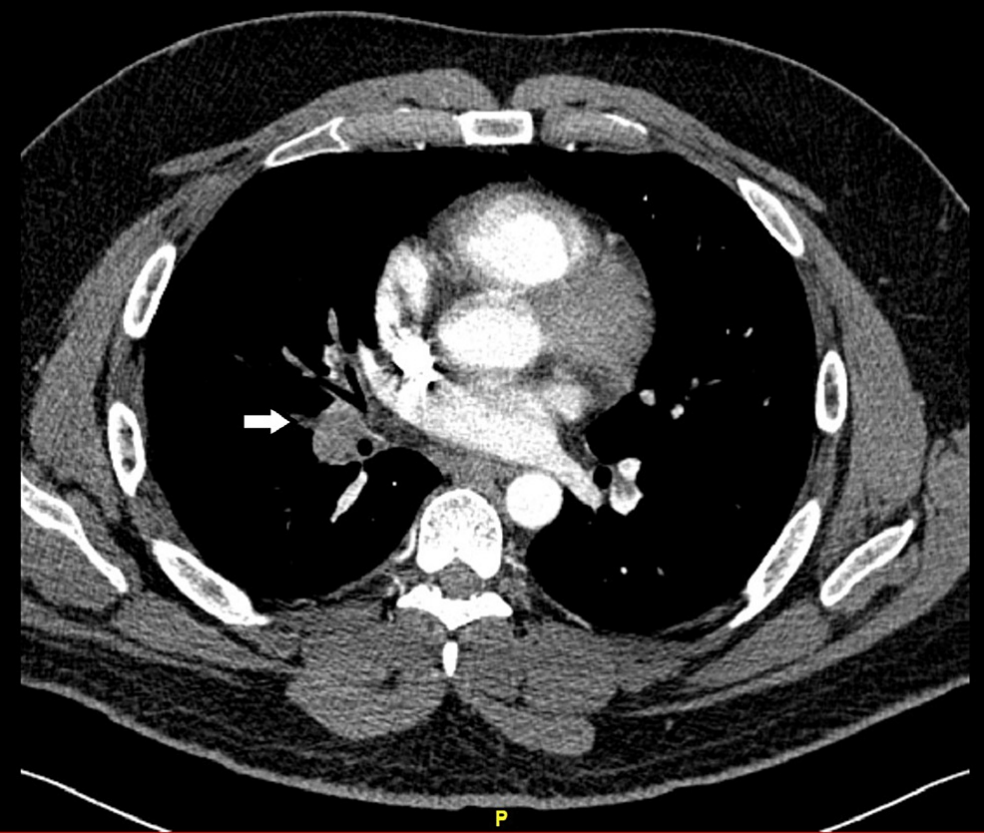
Arrow shows a filling defect in the right pulmonary artery consistent with pulmonary embolus.

An EKG was subsequently ordered and was normal without the S1 Q3T3 pattern. The complete blood count (CBC) and basic metabolic panel (BMP) were unremarkable. Given clot burden and reports of shortness of breath, computer tomography with angiography (CTA) of the chest was ordered. The CTA showed extensive acute pulmonary emboli involving the central, bilateral segmental, and subsegmental pulmonary arteries (Figure 2).

There was no evidence of heart strain. A filling defect in the internal jugular was noted and indeterminate for thrombus. On further questioning, he reported that his father was also on TRT at the time he developed a DVT. The patient now relayed he was also on TRT for low testosterone levels for a year, but he had recently discontinued his testosterone replacement therapy several weeks before when he initially had leg pain.

The patient was admitted to the hospital in stable condition after receiving a heparin bolus and infusion based on hospital-driven VTE dosing. He was subsequently discharged the following day on oral Eliquis for hematology follow-up.

## Discussion

There is conflicting evidence when reviewing the literature regarding the significance of TRT therapy and the development of VTE. Prior research from the 1990s has shown that androgens can increase the human platelet thromboxane A2 receptor density and formation [6]. In January 2014, the Food and Drug Administration issued a warning regarding the use of TRT due to the increased risk of stroke and myocardial infarction [7,8]. The Harvard Medical School issued a statement directly citing an increased risk for blood clots when using testosterone replacement therapy for low levels [8]. Dietary herbal supplements designed to enhance testosterone levels may also increase the risk of VTE due to hormone-induced polycythemia [9]. In a case report by Nguyen et al., the authors noted the increasing frequency of hormone supplementation even in men without medical conditions causing low testosterone levels [9]. With concerns about increasing prescriptions for TRT, a retrospective study was published in 2016 addressing the specific concern for TRT-induced VTE and reported that there was no correlation between TRT and VTE [10]. This study excluded women, men without documented prior levels, those with normal testosterone levels, or those with a history of VTE [10]. A second retrospective analysis was performed in 2018 reviewing the risk of TRT and VTE, and once again, this review by Houghton et al. found no correlation [11]. Corona et al. also reviewed epidemiological studies analyzing the link between TRT and cardiovascular disease and VTE in men who were prescribed testosterone for hypogonadism, concluded that no correlation was not present, and stated that any association was in men with previously undiagnosed thrombophilia [12].

More recent studies using meta-analysis on men with low testosterone levels who used TRT found that the observational data did not show a correlation; however, the review showed a need for more research as the analysis showed a lack of randomized controlled data [7]. Most recently, the TRAVERSE trial showed that TRT in men with hypogonadism with preexisting cardiovascular risk was non-inferior to a placebo [13]. Androgen use however is not limited to hypogonadism, but also by bodybuilders and non-athletes to enhance appearance and performance [3]. Menakuru et al. cited the expanded use of androgens and some association with a pulmonary embolus, but they stated that the relationship was poorly defined and risks of liver toxicity, polycythemia, cardiac disease, acne, gynecomastia, and testicular atrophy are the main risks identified [3]. Furthermore, the case study Menakuru et al. reviewed was the illicit use of testosterone for muscle enhancement, which was not the history with the patient in our case [3].

Earlier reports from decades prior favored a correlation between TRT and VTE. However, after a review of the recent literature, the conclusions favor a lack of correlation between TRT and VTE in the setting of hypogonadism but acknowledge that there could be more risk in the population who uses androgens for performance enhancement [3]. In 2019, Walker et al. published a study looking for the correlation of VTE in men with and without hypogonadism who are on TRT. This study again acknowledges that recent studies show conflicting results regarding association [2]. They concluded a correlation between TRT in men with and without hypogonadism with increased risk in younger males [2]. The case study above shows a high clot burden in a young adult male with treated hypogonadism. The amount of clot burden and family history would indicate a correlation between TRT and VTE, supporting Walker et al. in their conclusions.

## Conclusions

There is conflicting literature on risk factors associated with TRT and VTE. This case report combined with other reports indicates that this may need additional research to address the correlation of TRT with VTE, including replacement therapy and performance-enhancing use. With the increasing incidence of TRT and transgender applications, this case report shows the need for more studies to determine if there is a positive correlation between VTE and TRT in all patient populations, with and without hypogonadism. Clinicians should be aware of the potential association of TRT with VTE, acknowledging that diagnostic risk tools recognize estrogen but do not specifically include testosterone as a risk factor. This case report supports the recent study by Walker et al. and questions whether our current VTE risk analysis tools should be revised.
